# Métastase caverneuse d’une tumeur de vessie

**DOI:** 10.11604/pamj.2017.28.60.13244

**Published:** 2017-09-21

**Authors:** Mohammed Alae Touzani, Othmane Yddoussalah

**Affiliations:** 1 Université Mohammed 5, Faculté de Médecine et de Pharmacie de Rabat, Hopital Ibn Sina, Service d’Urologie B, Maroc

**Keywords:** Métastase, tumeur vésicale, corps caverneux, Metastasis, bladder tumor, corpora cavernosa

## Abstract

The incidence of cavernous metastases after radical treatment for bladder tumor is exceptional and is approximately 1%. The mean time between surgery and the occurrence of metastases is 8 months. In some cases they occur after 10 years. Diagnosis is confirmed by biopsy, which shows their urothelial origin. Here we report the case of 56-year old patient, with a history of smoking, having undergone total cystoprostatectomy in January 2017, who presented 2 months later with induratio penis plastica with no other sign associated. Priapism and gangrene of the penis of infectious origin have been suspected but, given the patient's clinical condition, the more likely diagnosis was cavernous metastasis. On two occasions the patient refused biopsy: during two consultations at 15 days interval (A, B). The patient accepted to undergo biopsy during the 3^th^ consultation (C, D). Biopsy confirmed the diagnosis of cavernous metastasis.

## Image en médecine

L'incidence d'apparition de métastases caverneuses après traitement radical d'une tumeur de vessie est exceptionnelle et est de l'ordre de 1%. Le délai d'apparition est en moyenne de 8 mois après la chirurgie, avec certains cas ou il arrive à 10 ans. Le diagnostic est confirmé par la biopsie qui met en évidence l'origine urothéliale. Nous rapportons ici le cas d'un patient de 56 ans, tabagique, ayant bénéficié en Janvier 2017 d'une cystoprostatectomie totale, qui consulte 2 mois plus tard pour une induration de la verge, sans autre signe associé. Le priapisme et la gangrène de la verge d'origine infectieuse ont été évoqués mais vu le contexte clinique, le diagnostic de métastase caverneuse était le plus probable. Le patient a refusé à deux reprises une biopsie, sur deux consultations à 15 jours d'intervalle (A, B). Une biopsie a été acceptée par le patient à la 3^ème^consultation (C, D), elle a confirmé le diagnostic de métastase caverneuse.

**Figure 1:**
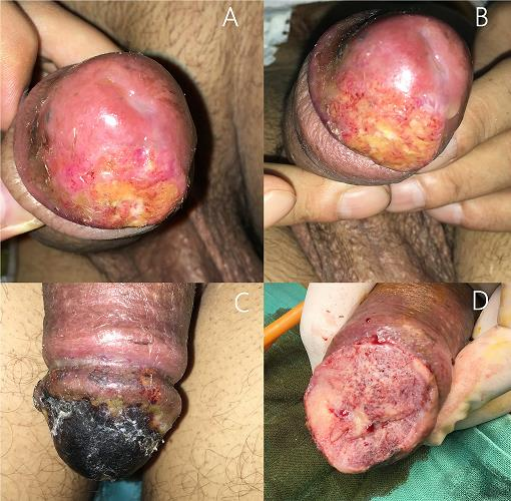
Métastase caverneuse

